# Formation of DNA Adducts by Ellipticine and Its Micellar Form in Rats — A Comparative Study

**DOI:** 10.3390/s141222982

**Published:** 2014-12-03

**Authors:** Marie Stiborova, Zuzana Manhartova, Petr Hodek, Vojtech Adam, Rene Kizek, Eva Frei

**Affiliations:** 1 Department of Biochemistry, Faculty of Science, Charles University, Albertov 2030, CZ-128-40 Prague 2, Czech Republic; E-Mails: zumicka@yahoo.com (Z.M.); hodek@natur.cuni.cz (P.H.); 2 Department of Chemistry and Biochemistry, Faculty of Agronomy, Mendel University in Brno, Zemedelska 1, CZ-613-00 Brno, Czech Republic; E-Mails: vojtech.adam@mendelu.cz (V.A.); kizek@sci.muni.cz (R.K.); 3 Division of Preventive Oncology, National Center for Tumor Diseases, German Cancer Research Center (DKFZ), Im Neuenheimer Feld 280, 69120 Heidelberg, Germany; E-Mail: evafrei@t-online.de

**Keywords:** ellipticine, ellipticine-derived DNA adducts, ^32^P-postlabeling imaging, nanoparticles, micelles

## Abstract

The requirements for early diagnostics as well as effective treatment of cancer diseases have increased the pressure on development of efficient methods for targeted drug delivery as well as imaging of the treatment success. One of the most recent approaches covering the drug delivery aspects is benefitting from the unique properties of nanomaterials. Ellipticine and its derivatives are efficient anticancer compounds that function through multiple mechanisms. Formation of covalent DNA adducts after ellipticine enzymatic activation is one of the most important mechanisms of its pharmacological action. In this study, we investigated whether ellipticine might be released from its micellar (encapsulated) form to generate covalent adducts analogous to those formed by free ellipticine. The ^32^P-postlabeling technique was used as a useful imaging method to detect and quantify covalent ellipticine-derived DNA adducts. We compared the efficiencies of free ellipticine and its micellar form (the poly(ethylene oxide)-*block*-poly(allyl glycidyl ether) (PAGE-PEO) block copolymer, P 119 nanoparticles) to form ellipticine-DNA adducts in rats *in vivo*. Here, we demonstrate for the first time that treatment of rats with ellipticine in micelles resulted in formation of ellipticine-derived DNA adducts *in vivo* and suggest that a gradual release of ellipticine from its micellar form might produce the enhanced permeation and retention effect of this ellipticine-micellar delivery system.

## 1. Introduction

Ellipticine (5,11-dimethyl-6*H*-pyrido(4,3-*b*)carbazole) and its derivatives are efficient anticancer compounds that function through multiple mechanisms participating in cell cycle arrest and initiation of apoptosis (for a summary see [1–6]). Ellipticine was found: (i) to arrest cell cycle progression due to modulation of levels of cyclinB1 and Cdc2, and phosphorylation of Cdc2 in human mammary adenocarcinoma MCF-7 cells; (ii) to initiate apoptosis due to formation of toxic free radicals, stimulation of the Fas/Fas ligand system and modulation of proteins of Bcl-2 family in several tumor cell lines; and (iii) to induce the mitochondria-dependent apoptotic processes (for a summary see [3,4]). The predominant mechanisms of ellipticine's biological effects were suggested to be (i) intercalation into DNA [5–7] and (ii) inhibition of topoisomerase II [3–6]. Further, we showed that this antitumor agent forms covalent DNA adducts after its enzymatic activation with cytochromes P450 (CYP) and peroxidases [1–4,8–13], suggesting an additional DNA-damaging effect of ellipticine. The ^32^P-postlabeling assay was found to be a useful method to image the covalent ellipticine-derived DNA adducts [1–4,8–13].

Of the CYP enzymes investigated, human CYP3A4 followed by CYP1A1 and 1B1 are the most active enzymes oxidizing ellipticine to 12-hydroxy- and 13-hydroxyellipticine, the reactive metabolites that dissociate to ellipticine-12-ylium and ellipticine-13-ylium species which form two major covalent DNA adducts [3,8–14]. The CYP1A isoforms also efficiently form the other ellipticine metabolites, 7-hydroxy- and 9-hydroxyellipticine, which are the detoxication products. Recently we have found that cytochrome b_5_ alters the ratio of ellipticine metabolites formed by CYP1A1, 1A2 and 3A4. While the amounts of the detoxication metabolites (7-hydroxy- and 9-hydroxyellipticine) were either decreased or not changed with added cytochrome b_5_, 12-hydroxy-, 13-hydroxyellipticine and ellipticine *N*^2^-oxide increased considerably. The change in amounts of metabolites resulted in an increased formation of covalent ellipticine-DNA adducts measured by the ^32^P-postlabeling method [11–13], one of the DNA-damaging mechanisms of ellipticine antitumor action [2,3,13]. In addition, we found that levels of the DNA adduct formed by 13-hydroxyellipticine also increased if this ellipticine metabolite was conjugated with sulfate or acetate by human sulfotransferases 1A1, 1A2, 1A3 and 2A1, or *N*,*O*-acetyltransferases 1 and 2 [11,13].

**Figure 1.**
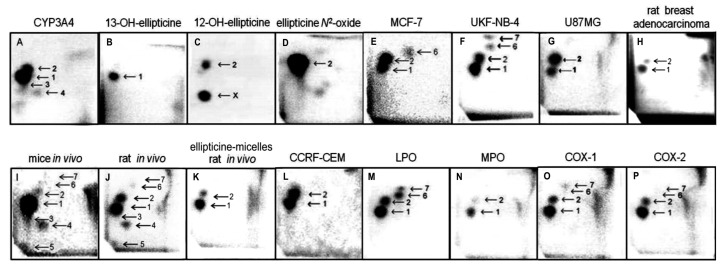
Autoradiographic profiles of ellipticine-derived DNA adducts analyzed with the ^32^P-postlabeling assay. Adduct profiles obtained from calf thymus DNA reacted with ellipticine and CYP3A4 [8] (**A**); from calf thymus DNA reacted with 13-hydroxyellipticine [8] (**B**); 12-hydroxyelipticine [9] (**C**); ellipticine *N*^2^-oxide [8] (**D**); from DNA of breast adenocarcinoma MCF-7 cells [18] (**E**); neuroblastoma UK-NB-4 cells [20] (**F**) and glioblastoma U87MG cells [22] (**G**) treated with 10 μM ellipticine; from DNA of breast adenocarcinoma of Wistar rats treated *i.p.* with 4 mg ellipticine per kilogram body weight [3] (**H**); from liver DNA of C57BL/6 mice treated *i.p.* with 10 mg ellipticine per kilogram body weight [16] (**I**); from liver DNA of Wistar rats treated *i.p.* with 10 mg ellipticine per kilogram body weight (present study) (**J**); from liver DNA of Wistar rats treated *i.p.* with 10 mg ellipticine in micelles per kilogram body weight [present study] (**K**) and CCRF-CEM cells [19] (**L**) treated with 10 μM ellipticine, from calf thymus DNA reacted with ellipticine and bovine lactoperoxidase (LPO) [9] (**M**); human myeloperoxidase (MPO) [9] (**N**); ovine cyclooxygenase [9] (COX)-1 (**O**) and human COX-2 [9] (**P**); Adduct spots 1–7 correspond to the ellipticine-derived DNA adducts. Besides adduct 2 formed by 12-hydroxyellipticine (**C**), another strong adduct (spot X in panel C), which was not found in any other activation systems or *in vivo* was generated.

The same ellipticine-derived DNA adducts that were found in *in vitro* incubations of ellipticine with DNA and enzymes activating this drug, were generated also *in vivo*, in several tissues of mice and rats exposed to ellipticine. The ellipticine-DNA adducts were also found in several cancer cell lines and in DNA of rat mammary adenocarcinoma *in vivo* [3,15–24] (Figure 1).

There are, however, several phenomena that can cause a limited usage of ellipticine and/or its limited anticancer efficiencies. This antineoplastic agent exhibits also severe adverse toxic effects, including nephrotoxicity, hemolysis, xerostomia, hypertension, nausea and vomiting (for a review see [13]). The question, therefore, arises how to eliminate its toxic side effects as well as to utilize novel knowledge on their bio-activation in cancer cells to potentiate their anticancer efficiencies in these cancer cells. Hence, the studies of our laboratory target on development of efficient and reliable methods for targeted delivery of ellipticine (and/or other anticancer drugs) as well as on preparation of this drug in the forms that exhibit lower side effects and leads to an increase in their anticancer effects. One of the aims is to develop nanocarriers that will contain this drug. The advantages of the drug delivery performed by nanocarriers such as iron oxides, gold, biodegradable polymers, dendrimers, and lipid based carriers (*i.e.*, liposomes or micelles) have been extensively investigated (for a summary see [25–27]). Even though the experiments to prepare encapsulated ellipticine have already been carried out, knowledge on its cytotoxicity to cancer cells and its fate in organisms are still scarce [28–41].

In this study, we utilized a micellar form of ellipticine to study a comparison between the biodistribution of this drug form and free ellipticine to reach the tissues in which the formation of covalent ellipticine-derived DNA adducts are generated. Polymeric micelles that attract an increasing interest in contemporary drug research because they could be used as a very efficient drug delivery system [42,43] were employed for such a study. Polymeric micellar drug delivery systems (MDDSs) of core-shell architecture based on amphiphilic AB diblock or ABA triblock copolymers possess numerous advantages [42–44]. One of them is the fact that they improve solubility and bioavailability of hydrophobic drugs that are poorly soluble or insoluble in water [42–44]. This is also the case of a hydrophobic molecule of ellipticine that is poorly soluble in water. Micelles with biocompatible hydrophilic shell show low uptake by the reticuloendothelial system even if they have a nonbiocompatible core [45,46] and significantly protect the incorporated drug from fast degradation, blood clearance and elimination from the body. Micelles are, due to thermodynamic reasons, of very narrow size distribution, which is very important for their biodistribution, and they behave like single molecules in the cases where biodistribution is dependent on molecular weight. This is advantageous especially in the case of drug delivery systems designed for delivery of cancerostatics where high molecular weight together with biocompatibility of the system is often beneficial to achieve effective passive targeting into solid tumour tissue due to leaky vasculature and reduced or missing lymphatic drainage in these tissues [the so-called Enhanced Permeation and Retention (EPR) effect] [44–48].

In this work, we used the poly(ethylene oxide)-*block*-poly(allyl glycidyl ether) (PAGE-PEO) block copolymer (P 1191 nanoparticles) [44] to prepare a micellar form of ellipticine and investigated its biodistribution among the tissues of a model organism, Wistar rats, which might, to some extent, mimic the fate of ellipticine in humans [10,13,15,16]. The formation of ellipticine-derived DNA adducts as the biological end-point of the pharmacological and genotoxic effects of this drug, mediated by free ellipticine and its micellar form, measured with the ^32^P-postlabeling assay as the imaging method [1–3,8–24], were employed to determine their biodistribution in rats *in vivo*.

## 2. Experimental Section

### 2.1. Chemicals and Materials

Ellipticine, NADPH, and calf thymus DNA were from Sigma Chemical Co. (St. Louis, MO, USA). Enzymes and chemicals for the ^32^P-postlabeling assay were obtained from sources described [1]. Other chemicals were of analytical grade or better. The poly(ethylene oxide)-*block*-poly(allyl glycidyl ether) (PAGE-PEO) block copolymer (P 1191 nanoparticles), prepared and characterized as described previously [44,45], were a gift of Dr. M. Hruby (Institute of Macromolecular Chemistry AS CR, Prague, Czech Republic). Ellipticine, dissolved in dimethy sulfoxide (DMSO) into a saturation concentration, was gradually added into a water solution of polymeric nanoparticles P119 and the mixture incubated at 23 °C for 24 h. A concentration of a non-covalently bound ellipticine in a hydrophobic core was determined spectrophotometrically (at 310 nm). Free micelles (nanoparticles P119) were also labeled with ^125^I as described [49].

### 2.2. Ellipticine Release from Micelles In Vitro

Stability of ellipticine-micelles and ellipticine release from these micelles were determined by a dialysis method [46,50] after their incubation in 0.1 M sodium phosphate buffer pH 5.0 and 7.4 at 25 °C and measured spectrophometrically (at 310 nm).

### 2.3. Ellipticine Release from Micelles and Its Transfer into Rat Liver Microsomes In Vitro

Release of ellipticine from ellipticine-micelles in the presence of liver microsomes (a cellular membrane fraction formed from broken endoplasmic reticulum) of Wistar rats [1,10] and its transfer into these subcellular membrane particles were investigated in incubation mixtures of these particles in 0.1 M sodium phosphate buffer pH 7.4 for 0, 9, 17, 24, 48 h and 2 weeks at 4 °C with rat liver microsomes. After these incubations, aliquots of the mixtures containing 0.075 μmol ellipticine were transferred into the mixtures of 50 mM phosphate buffer pH 7.4 containing rat hepatic microsomes (1 mg microsomal protein), 1 mM NADPH (a cofactor of a CYP-mediated enzyme system), and 1 mM calf thymus DNA (a total volume of the mixture was 0.75 mL) and further incubated for 1 h at 37 °C. Analogously, the same amounts of ellipticine as a free compound was incubated with DNA, microsomes and NADPH for 1 h at 37 °C. After incubation, DNA was isolated from the mixtures and ellipticine-derived DNA adducts were analyzed. DNA was isolated from the reaction mixtures by the phenol-chloroform extraction as described [1].

### 2.4. Treatment of Rats with Free Ellipticine and Ellipticine-Micelles for Ellipticine-Derived DNA Adduct Analyses

The study was conducted in accordance with the Regulations for the Care and Use of Laboratory Animals (311/1997, Ministry of Agriculture, Prague, Czech Republic), which is in compliance with the Declaration of Helsinki. Animals were acclimatized for five days and maintained at 22 °C with a 12 h light/dark period. Standardized diet and water were provided *ad libitum*. Groups of five weeks old male Wistar rats (∼150 g, *n* = 3/group) were treated *i.p.* with one dose of 4 or 10 mg of ellipticine (dissolved in water in the presence of acetic acid, for details, see [10]) or the same amounts of ellipticine present in ellipticine-micelles in a water solution per kg body weight. Animals in the control groups received vehicle only. Animals were killed three days after the treatment. Liver, spleen, kidney, heart, lung and brain were removed after sacrifice, frozen in liquid nitrogen and stored at −80 °C until analysis. DNA from organs was isolated by a standard phenol/chloroform extraction method [10].

### 2.5. ^32^P-Postlabeling of Ellipticine-Derived DNA Adducts

DNA samples isolated both from the incubations (see Section 2.2.) and from the organs of rats treated with ellipticine or ellipticine-micelles (see Section 2.3.) were analyzed for the presence of ellipticine-derived DNA adducts by the nuclease P1 version of the ^32^P-postlabeling method as described [1,8–10]. Samples of calf thymus DNA incubated with 13-hydroxy- and 12-hydroxyellipticine [8,9] analyzed by the same method were used to compare DNA adduct spot patterns. Chromatographic conditions for thin layer chromatography (TLC) on polyethylenimine-cellulose plates (10 cm × 20 cm; Macherey-Nagel, Düren, Germany) were: D1, 1.0 M sodium phosphate, pH 6.8; D3: 3.5 lithium formate, 8.5 M urea, pH 3.5; D4, 0.8 M lithium chloride, 0.5 M Tris-HCl, 8.5 M urea, pH 8; D5, 1,7 M sodium phosphate, pH 6. After chromatography TLC sheets were scanned using a Packard Instant Imager (Dowers Grove, IL, USA) and DNA adduct levels (RAL, relative adduct labeling) were calculated as described [1,8–10].

### 2.6. Statistical Analyses

For statistical data analysis we used Student's *t*-test. All *P*-values are two-tailed and considered significant at the 0.05 level.

## 3. Results and Discussion

### 3.1. Preparation of the Micellar Form of Ellipticine

Initially, a possibility of preparation of a micellar form of ellipticine as the micellar polymeric drug delivery system (MDDS) was investigated. Ellipticine was physically loaded into micelles formed from the poly(ethylene oxide)-*block*-poly(allyl glycidyl ether) (PAGE-PEO) block copolymer (P 119 nanoparticles) that were prepared and self-assembled into the micellar structure by Hruby and collaborators as described in their work [44,45]. The prepared micelles with narrow size distribution were characterized and used by these authors for hydrophobic entrapping of some hydrophobic compounds such as an azo dye Sudan III and an anticancer drug doxorubicin [44,45]. In the present study, where ellipticine dissolved in DMSO was gradually added into a water solution of polymeric nanoparticles P 119 and the mixture incubated at 23 °C for 24 h, the micelles containing 0.595 mg ellipticine per mL under the concentration of polymer of 25.9 mg per mL of water were prepared.

### 3.2. Ellipticine Release from Micelles In Vitro

**Figure 2.**
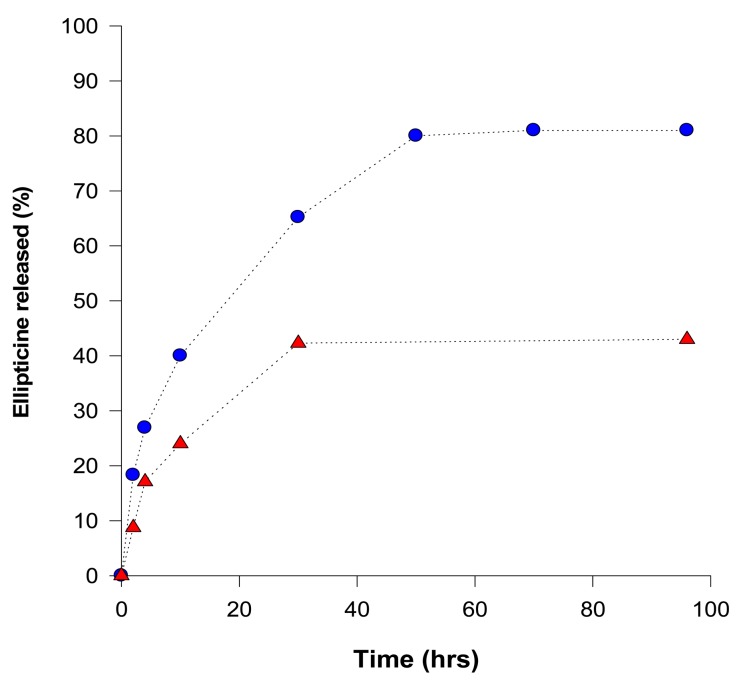
Ellipticine release from ellipticine-micelles at pH 5.0 (●) and 7.4 (▴). Values are given as means of two parallel experiments.

Release of ellipticine from micelles was analyzed by a dialysis method [46,50] and concentrations of ellipticine evaluated by UV absorption spectroscopy (using an ellipticine absorption maximum at 310 nm). Release of ellipticine from the micelles, measured by dialysis, proceeded in two stages: an initial rapid release was followed by a stage of slow and long-lasting release of ellipticine (Figure 2). At pH 7.4, during 96 h more than 43% of ellipticine was released. Acceleration of ellipticine release was obtained by lowering the surrounding pH from 7.4 to 5.0 (the pH value similar to pH of the cancer cells); more than 81% ellipticine was released after 96 h incubation at pH 5.0, suggesting a pH-sensitive release of ellipticine from the micelles (Figure 2). Moreover, this finding demonstrates that ellipticine as a hydrophobic base compound is easily released from the micelle into the water environment under the acidic conditions.

### 3.3. Ellipticine Release from Micelles and Its Transfer into Rat Liver Microsomes In Vitro

**Figure 3.**
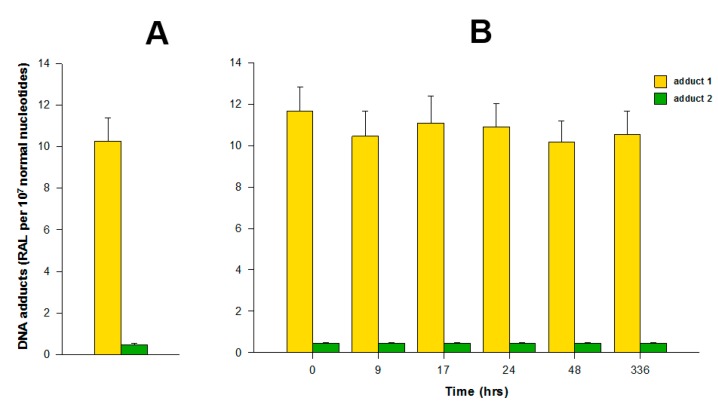
Ellipticine release from ellipticine-micelles and its transfer into rat liver microsomes. Efficiencies of transfer was measured by formation of ellipticine-derived DNA adducts (adducts 1 and 2 generated by 13-hydroxyellipticine and 12-hydroxyellipticine, respectively, see Figure 1) formed from the released ellipticine activated by rat liver microsomes. Formation of ellipticine-derived DNA adducts by free ellipticine (**A**) and released ellipticine (**B**) activated with rat liver microsomes were determined by the ^32^P-postlabeling method. Data shown are means ± S.D. from three parallel experiments. RAL, relative adduct labeling.

The incubations of both free elllipticine and ellipticine-micelles with rat liver microsomes were employed to analyze the transfer of both forms of this drug into these membrane particles. Microsomes incubated with free ellipticine and its micellar form in the presence of NADPH that acts as a cofactor of a CYP-mediated enzyme system present in microsomes were capable of generating ellipticine-derived DNA adducts detected by the ^32^P-postlabeling assay [1,8–10]. Two major adducts 1 and 2 (see adduct spots 1 and 2 in Figure 1), which are formed from two ellipticine metabolites, 13-hydroxyellipticine and 12-hydroxyellipticine, respectively (Figure 1), were generated. The levels of adducts were quantified by determining the ^32^P radioactivity of the adducts and expressed as relative adduct labeling (RAL) (Figure 3). Essentially the same levels of ellipticine-DNA adducts were formed in the incubations of ellipticine and ellipticine-micelles, 10.2 ± 1.2 and 11.6 ± 1.3 adducts per 10^7^ normal deoxynucleotides, respectively (Figure 3). The same results were found even when ellipticine-micelles were incubated with microsomes for 9, 17, 24, 48, and 336 h in 50 mM sodium phosphate buffer pH 7.4 at 37 °C; ellipticine is released from micelles and enters the membrane of microsomes where is further oxidized to 13-hydroxyellipticine and 12-hydroxyellipticine by the CYP enzymes present in this microsomal membrane, thereby forming the two DNA adducts. These results indicate that both free ellipticine and ellipticine present in micelles are capable of entering the biological membrane, namely the membrane of endoplasmic reticulum. They also suggest that ellipticine in a micellar form might when is transported to the target cells enters these cells or their compartments; the mechanism of the ellipticine enter into the cells seems to be the transfer of free ellipticine released from the micelles across the membrane.

### 3.4. Distribution of the PAGE-PEO Block Copolymer (P 1191 Nanoparticles) in Rats In Vivo

Using micelles (P 119 nanoparticles) labeled with ^125^I, a tissue distribution of these micelle nanoparticles in rats *in vivo* was investigated. Two days after *i.p.* administration of ^125^I-labeled micelles to rats (135 Bq/kg body weight), the ^125^I radioactivity was detectable in all tested organs indicating well distribution of these micelles in rats by their transport from peritoneum into the different rat organs. The highest levels of radioactivity were found in spleen (1650 Bq/g), followed by those in lung (1580 Bq/g), liver (850 Bq/g), heart (500 Bq/g) and kidney (380 Bq/g). The lowest but detectable levels of the ^125^I radioactivity were found in brain (100 Bq/g). The detection of ^125^I radioactivity in brain reveals the ability of micelles to overcome the hematoencephalic barrier (the blood-brain barrier). This is an important feature, because the possibility to treat central nervous system disorders is strongly limited by the poor access of many therapeutic agents to the target tissues. This is, namely, mainly due to the presence of the blood-brain barrier, formed by a complex interplay of endothelial cells, astrocyte and pericytes, through which only selected molecules can passively diffuse to reach central nervous system [51]. This finding emphasizes that a micellar form of ellipticine might probably be employed to treat the brain tumors, treatment of which by several cytostatics is usually limited because of strict selectivity of the hematoencephalic barrier. The high levels of ^125^I radioactivity in spleen and lung seemingly follow from the high circulating blood to these tissues relative to other tested organs. The relative low levels of the ^125^I radioactivity detected in kidney can be caused by lowering filtration of micelles to this organ. This is an important phenomenon, because drug-carries are usually used to prolong their circulation in blood. A relative high molecular weight of micelles protects the whole drug delivery system against their fast elimination from organisms. Lowering filtration of micelles in kidney is suitable for prolongation of drug retention in organisms.

### 3.5. Ellipticine-Derived DNA Adduct Formation by Free Ellipticine and Ellipticine-Micelles in Rats In Vivo

In order to image the ellipticine-derived DNA adducts formed in various organs of male Wistar rats treated *(i.p.*) with a single dose of free ellipticine or ellipticine in micelles (4 and 10 mg/kg body weight), the nuclease P1 version of the ^32^P-postlabeling assay [1,8–10] was again used. Two of the ellipticine-DNA adducts (spots 1 and 2 in Figure 1) formed from two ellipticine metabolites, 13-hydroxyellipticine and 12-hydroxyellipticine [3,4,8,9,11,13], were found in DNA of all tested tissues of rats exposed to these ellipticine forms.

Besides these adducts, up to five additional adducts were detected in DNA of liver, kidney, lung, spleen and heart treated with free ellipticine (see Figure 1J for liver DNA of rats treated with ellipticine). Adduct spot 3, migrating close to the major adduct 1 and therefore partially overlapping with this adduct (Figure 1), was detected in liver, kidney, lung, and heart of rats. Adduct spots 4 and 5, located on a diagonal zone on the TLC plates (spot 5 close to the origin) were found in liver, lung, kidney, and heart of rats, while adduct spots 2–4 were also generated as minor adducts in *in vitro* experiments using rat and human hepatic microsomes or human CYP enzymes as activating systems [1,3,8] (see Figure 1A for CYP3A4), adduct spot 5 has never been detected in these *in vitro* enzymatic systems. Two minor adducts (spots 6 and 7 in Figure 1) were detected in DNA of liver, kidney, lung and spleen of rats treated with 10 mg/kg of body weight of ellipticine. These adducts are probably generated from ellipticine mainly by peroxidase activation (Figure 1 M, O, P for lactoperoxidase, COX-1, COX-2, respectively) [9]. Since only low amounts of the minor adducts (spots 3, 4, 6 and 7 in Figure 1) were formed, their further structural characterization was not possible [2–4,13]. No adduct spots were detected in DNA isolated from organs of rats treated with solvent only.

**Figure 4.**
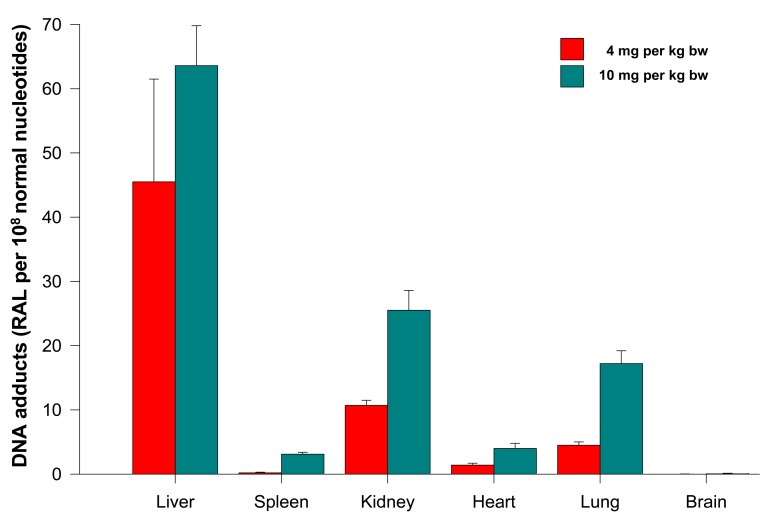
Ellipticine-derived DNA adducts (total levels of DNA adducts) formed in rats treated with free ellipticine (4 and 10 mg per kg body weight (bw)). Data shown are means ± S.D. from three rats. RAL, relative adduct labeling.

The levels of adducts were again quantified by determining the ^32^P radioactivity of the adducts and expressed as RAL (Figure 4). As shown in Figure 4, the highest total DNA binding was found in liver, followed by kidney, lung, heart, spleen and brain of rats treated at both ellipticine dosages, which essentially correspond to expression and activities enzymes oxidizing ellipticine to 13-hydroxyellipticine and 12-hydroxyellipticine such as CYP3A, 2C and 1A [15] together with cytochrome b_5_, the protein that influence their activities to form higher amounts of these metabolites [11,12], and/or peroxidases [9]. The total DNA adduct levels were in a range from 0.02 to 63.6 adducts per 10^8^ nucleotides in the excised rat organs. The lowest amounts of ellipticine-derived DNA adducts were found in brain (0.02 ± 0.01 and 0.05 ± 0.01 adducts per 10^8^ normal deoxyribonucleotides in brain DNA of rats treated with 4 and 10 mg ellipticine/body weight, respectively). Similar to the total adduct levels, the highest amount of the major adduct (spot 1) was detected in liver of rats. In contrast to this adduct, the highest levels of adduct 2 were found in kidney of rats treated with 10 mg of ellipticine per kg of body weight (data not shown). An increase in dose of ellipticine resulted in an increase in total ellipticine-DNA adducts formed in all organs analyzed (Figure 4).

**Figure 5.**
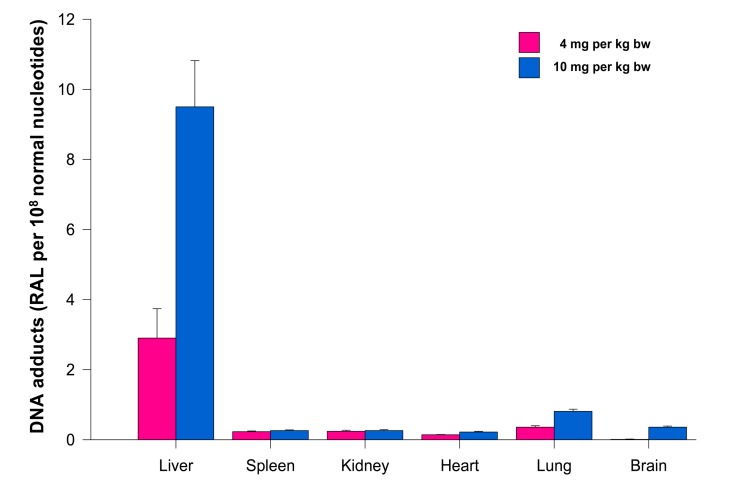
Ellipticine-derived DNA adducts (total levels of DNA adducts) formed in rats treated with ellipticine-micelles (4 and 10 mg ellipticine in micelles per kg body weight (bw)). Data shown are means ± S.D. from three rats.

Treatment of rats with ellipticine in micelles (4 and 10 mg ellipticine in micelles per kg body weight) resulted in formation of DNA adducts 1 and 2 in liver (Figure 1K), lung and kidney of rats treated with both dosages of ellipticine-micelles, but the adduct 2 was not detectable in spleen, heart and brain. The additional minor adducts (adduct spots 3–7 in Figure 1) were not detectable in any of the tested organs. Total levels of ellipticine-DNA adducts were again highest in liver, followed by lung > brain > kidney > spleen > heart of rats treated at both doses of ellipticine-micelles (Figure 5). The amounts of ellipticine-derived DNA adducts found in individual organs of rats treated with ellipticine-micelles did not parallel with the levels of ^125^I radioactivity in these organs of rats treated with micelles containing ^125^I. These findings indicate that not only the biodistribution of micelles in rat body, but also expression levels of enzymes activating ellipticine to metabolites binding covalently to DNA in these organs dictate the amounts of adducts formed in these organs. The levels of ellipticine-DNA adducts formed in rat tissues after their administration with ellipticine-micelles were one order of magnitude lower in most organs than in those of rats treated with free ellipticine (*p* < 0.001, different from levels of ellipticine-derived DNA adducts formed by free ellipticine; comparison was performed by *t*-test analysis), with an exception of brain, where levels of ellipticine-DNA adducts formed by administration of rats with ellipticine-micelles were higher than in DNA of brain of rats treated with free ellipticine (0.05 ± 0.01 and 0.35 ± 0.03 adducts per 10^8^ normal deoxyribonucleotides in brain DNA of rats treated with 10 mg of free ellipticine and ellipticine in micelles, respectively). This again demonstrates the ability of ellipticine-micelles to overcome the blood-brain barrier.

At least two reasons might account for the lower amounts of ellipticine-derived DNA adducts formed in most organs of rats treated with ellipticine in micelles. The lower levels of ellipticine-DNA adducts in rat organs might follow from gradual release of ellipticine from micelles causing its lower actual concentrations available for oxidative activation. This corresponds to the enhanced permeation and retention (EPR) effect of the ellipticine-micellar delivery system. Nevertheless, the lower levels of ellipticine-DNA adducts in rat tissues might also be caused by another feature, by the fact that a part of ellipticine (that is not bound in micelles covalently) is released from ellipticine-micelles already in peritoneum, where a relative high amount of lipocytes (fat cells) are present, and can bound the hydrophobic molecules of ellipticine. In order to resolve which of these explanations is more probable, the experiments employing *i.v.* administration of ellipticine in micelles should be performed. In addition, a study investigating a stability of ellipticine-micelles in the presence of fat cells should be carried out.

## 4. Conclusions

Utilizing the ^32^P-postlabeling assay found previously to be a suitable imaging method to detect and quantify ellipticine-derived DNA adducts formed *in vitro* and *in vivo*, their formation from free ellipticine and its micellar form (the poly(ethylene oxide)-*block*-poly(allyl glycidyl ether) block copolymer, P 119 nanoparticles) in rats *in vivo* was compared. The results demonstrate that treatment of rats with free ellipticine or this anticancer agent in micelles resulted in formation of ellipticine-derived DNA adducts in liver, spleen, kidney, heart, lung and brain of rats treated with these forms of ellipticine. The levels of ellipticine-DNA adducts formed in rat tissues after their administration with ellipticine-micelles were one order of magnitude lower in most organs than in those of rats exposed to free ellipticine, with an exception of brain, where levels of ellipticine-DNA adducts formed by ellipticine in micelles were higher than in DNA of brain of rats treated with free ellipticine. The lower levels of ellipticine-DNA adducts might indicate a gradual release of ellipticine from micelles that might produce the enhanced permeation and retention effect of the ellipticine-micellar delivery system. The results found in this study are the first finding showing the biodistribution of an anticancer drug ellipticine encapsulated in micelles *in vivo*. They also form the significant basis for the preparation of transporting nanocarriers suitable for the targeted delivery of ellipticine.
